# Composition, mineralization potential and release risk of nitrogen in the sediments of Keluke Lake, a Tibetan Plateau freshwater lake in China

**DOI:** 10.1098/rsos.180612

**Published:** 2018-09-26

**Authors:** W. W. Wang, X. Jiang, B. H. Zheng, J. Y. Chen, L. Zhao, B. Zhang, S. H. Wang

**Affiliations:** 1 National Engineering Laboratory for Lake Pollution Control and Ecological Restoration, Chinese Research Academy of Environmental Sciences, Beijing 100012, People's Republic of China; 2 College of Water Sciences, Beijing Normal University, Beijing 100875, People's Republic of China

**Keywords:** sediment, nitrogen speciation, mineralization, release flux, plateau freshwater lake, Keluke Lake

## Abstract

The lakes distributed in the Tibetan Plateau constitute a lake group with the highest altitude, largest lakes and largest area in the world and are important in global climate and environmental effects. Freshwater lakes in the Tibetan Plateau possess high ecological values and high vulnerability. The migration and transformation of nitrogen in sediments are critical to lake ecosystems, but information on sedimentary nitrogen in the freshwater lakes in the Tibetan Plateau is limited. A case study was conducted in Keluke Lake, China, to reveal the effects of sedimentary nitrogen on water quality in plateau freshwater lakes. Nitrogen speciation, mineralization potential and release flux were analysed through a sequential extraction method, waterlogged incubation experiment and Fick's first diffusion law, respectively. The content of total nitrogen (TN) was 1295.75–6151.69 mg kg^−1^, and 94.2% of TN was organic nitrogen (ON). The contents of three nitrogen fractions were in the order of hydrolysable nitrogen > residual nitrogen > exchangeable nitrogen. Ammonia nitrogen $\left(\text{N}{\text{H}}_{4}^{+}-\text{N}\right)$ was the main mineralization product, and hydrolysable ON was the most significant contributor. The sediments showed a great mineralization potential, with a potentially mineralizable nitrogen value of 408.76 mg N kg^−1^ of sediment, that was mainly affected by hydrolysable ammonium nitrogen. The $\text{N}{\text{H}}_{4}^{+}-\text{N}$ diffusion flux ranged from 24.14 to 148.75 mg m^−2^ d^−1^, and the sediments served as an internal nitrogen source. Nitrogen release from sediments was considerably influenced by exchangeable ammonia nitrogen. The sediments in Keluke Lake pose a potential nitrogen release risk and threaten the water quality of the lake. The total content, speciation, mineralization of ON and the release flux at sediment–water interface should be considered comprehensively to evaluate the effects of nitrogen in sediments to water quality.

## 1. Introduction

Nitrogen is a major nutrient in aquatic ecosystems and a driving factor of lake eutrophication associated with energy flow and material circulation in lake ecosystems [1,2]. Nitrogen in lakes originates from external (e.g. municipal sewage, industrial effluents, rainfall, runoff and soil leaching) and internal (e.g. aquatic plants, zooplankton and phytoplankton, and sediments) sources [3–5]. Large amounts of external pollutants (e.g. nutrients, metals and organic pollutants) are induced into natural water bodies by human activities [6]. These pollutants partly accumulate in sediments after a series of biochemical processes and cause sediment pollution.

Lake sediments are the primary source of pollutants (e.g. nutrients, heavy metals and organic contaminants) in water ecological ecosystems [7–9]. Lake sediments usually act as a sink for nitrogen [10]. The nitrogen in sediments can be released into the water column again under certain conditions (e.g. low pH, anaerobic condition and disturbance) [11,12]. In such a case, sediments act as a source and support the primary production of the lake ecosystem by nitrogen sediment flux. However, nitrogen in sediments exists in different forms and with varied mobility. Not all nitrogen fractions are bioavailable, and not all nitrogen in sediments can be released into water bodies. Numerous studies on the fractionation and bioavailability of nitrogen in lake sediments have shown that the mobility and bioavailability of nitrogen in sediments primarily depend on how nitrogen is bound to other elements rather than the total content alone [13]. Thus, the role of nitrogen in sediments and its contributions to lake eutrophication must be evaluated based on different nitrogen fractions rather than total nitrogen (TN) only. The nitrogen forms in lake sediments have been extensively studied [2,14–17]. These studies have provided valuable information on whether sediments act as an absorber or a source of nitrogen and have explored nitrogen cycling in lake ecosystems. However, previous studies focused on lowland plain lakes, and only a few have been conducted on plateau freshwater lakes with high altitudes. The impacts of sediments with high nitrogen load on the aquatic ecosystem of plateau freshwater lakes remain unclear.

The lakes distributed in the Tibetan Plateau constitute the largest lake group with the highest altitude lakes in the world. However, most of them are saltwater lakes. The plateau only has 51 freshwater lakes with an area larger than 1 km^2^. Plateau freshwater lakes have high ecological and economic values but fragile ecological environments. The water ecosystem is difficult to recover once it is destroyed. A study has shown that TN in the lake sediments of the Tibetan Plateau ranges from 0.26 to 10.84 g kg^−1^ [2], and the maximum value is higher than the severe effect level (4.80 g kg^−1^) regulated in the sediment quality evaluation standard set by Ontario Ministry of Environment and Energy [18], thus posing a great potential risk for lake ecosystems. The risk could even increase under the background of climate change [19]. Hence, studying the occurrence of characteristic and dynamic changes in nitrogen in sediments is crucial to understanding the impacts of sediments on freshwater lake ecosystems in the Tibetan Plateau.

Keluke Lake is located in the Tibetan Plateau, and it is the largest freshwater lake in Qaidam Basin. A previous study has shown that the water quality of Keluke Lake is decreasing; and TN, ammonia nitrogen $\left(\text{N}{\text{H}}_{4}^{+}\text{-N}\right)$ and chemical oxygen demand (COD) are the primary typical pollutants [20]. The average content of TN, $\text{N}{\text{H}}_{4}^{+}\text{-N}$ and COD increased from 1.11, 0.22 and 17.92 mg l^−1^ in 2014 to 1.54, 0.69 and 18.19 mg l^−1^ in 2015, respectively [20]. Although the content of COD has increased, it is still within Class III specified in the environmental quality standards for surface water of China (GSB3838–2002), which divides the surface water into five categories according to the environmental function and protection target of surface water. The content of total phosphorus (TP) was lower than the threshold value of Class II. The results of the research on the phosphorus pollution in sediments of Keluke Lake showed that the mean content of TP was 548.14 mg kg^−1^ and in mild contamination level. The release flux of phosphorus at the sediment–water interface ranged from −0.0002 to 0.0022 mg d^−1^ m^−2^, with a mean value of 0.0006 mg d^−1^ m^−2^, indicating that the release risk of phosphorus in sediments of Keluke Lake was low. However, the contents of TN and $\text{N}{\text{H}}_{4}^{+}\text{-N}$ changed from Class IV to Class V and from Class II to Class III, respectively. Thus, nitrogen is the main factor that affects the water quality of Keluke Lake. The remigration of nitrogen in sediments is closely related to the high nitrogen concentration in overlying water. To ascertain the characteristics of nitrogen fractions and estimate the potential contribution of sedimentary nitrogen to the water quality of Keluke Lake, we performed the following tasks. Firstly, a sequential extraction scheme was used to investigate the spatial distribution of nitrogen fractions in sediments. Secondly, the nitrogen pollution characteristics in sediments of Kelule Lake were compared with those of other typical lakes in China and other countries. Thirdly, a waterlogged incubation experiment was conducted in a laboratory to evaluate the nitrogen mineralization potential. Fourthly, a correlation analysis was performed between potentially mineralizable nitrogen (PMN) and different organic nitrogen (ON) fractions to determine the dominant contributors to nitrogen mineralization. Lastly, the nitrogen release flux was estimated to assess the influence of sediment nitrogen release on water quality. This study helps in understanding nitrogen cycling in plateau freshwater lakes.

## 2. Material and methods

### 2.1. Study area

Keluke Lake (37.25° N–37.33° N, 96.85° E–96.97° E) is located in Delhi, Haixi State, Qinghai Province, and belongs to Bay in River Basin. The lake has an area of 58.6 km^2^, an altitude of 2813.8 m, an average water depth ranging from 4 to 5 m (the maximum is 13.8 m) and a water volume of 1.67 × 10^8^ m^3^. Bayin River and Balegeng River are recharging water sources for Keluke Lake and lie in the northeast and northwest of the lake, respectively. Excess water in Keluke Lake will overflow into Tuosu Lake, which is in the southeast of Keluke Lake. Keluke Lake is the largest freshwater lake and the region with the highest biodiversity in Qaidam Basin [20]. The lake is crucial in maintaining the ecological safety and climate regulation of the local area.

### 2.2. Sample collection and treatment

**Figure 1.**
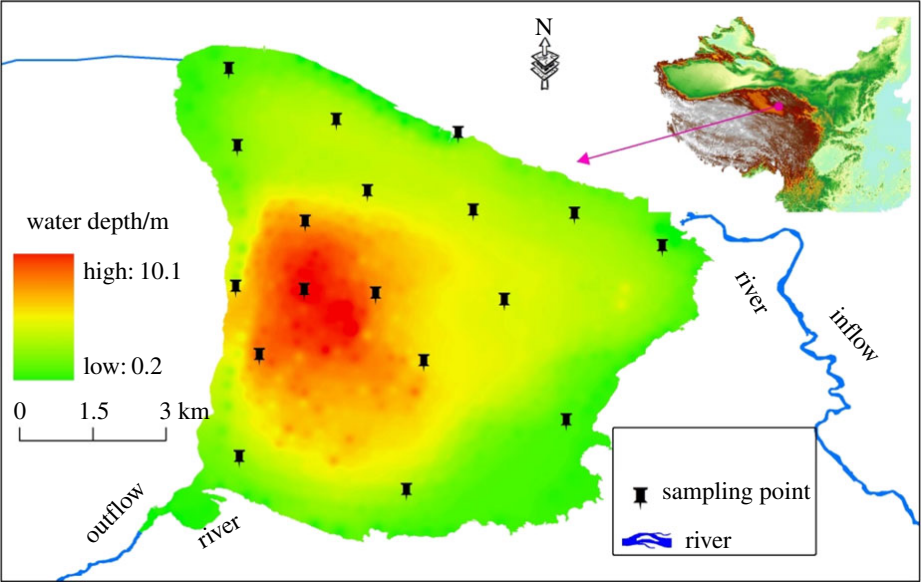
Sampling stations of surface sediment and overlying water samples in Keluke Lake.

Surface sediment and overlying water samples were collected from 18 sampling sites in June 2016 (figure 1). The accurate positions of all sampling sites were recorded by GPS. The sediment samples were collected with a grab sampler (04.30 Van Veen, Wildco). Three adjacent surface sediment samples were collected from each site and mixed evenly in the field. Stones and plant and animal residues in the sediments were removed, and the sediment samples were stored in clean polythene bags. The water samples were collected with an organic glass hydrophore and then stored in clean plastic tubs. All of the sediment and water samples were stored in iceboxes at 4°C before being transferred to the laboratory. A mount of evenly mixed fresh sediment was placed in 100 ml centrifuge tubes and centrifuged at 5000 r.p.m. for 15 min at 4°C. The supernatant was filtered through 0.45 μm Millipore filters (mixed cellulose ester membrane) to obtain interstitial water. The sediment samples were then dried with a vacuum freeze-drying equipment at −50°C and sifted through a sieve (100 mesh, 0.149 mm) before analysis. Analysis of the water samples was performed in 48 h. The contents of $\text{N}{\text{H}}_{4}^{+}\text{-N}$ in the overlying and interstitial water were determined according to the National Standard Method in China [21].

### 2.3. Analysis of nitrogen fractions

The nitrogen in the sediments was divided into three forms: exchangeable nitrogen (EN), acid hydrolysable nitrogen (HN) and residual nitrogen (RN). The nitrogen fractions were determined with the modified sequential extraction method of Wang *et al*. [22].

#### 2.3.1. Exchangeable nitrogen

EN was extracted by adding 100 ml of KCl (2 mol l^−1^) into 5 g of sediments. The mixture was shaken for 2 h and centrifuged at 5000 r.p.m. for 15 min. The supernatant was filtered through a 0.45 µm filter membrane. The residues were freeze dried and kept for HN extraction in the next step. EN in the sediments mainly contained ion-exchangeable $\text{N}{\text{H}}_{4}^{+}\text{-N}$ $\left(\text{E-N}{\text{H}}_{4}^{+}\text{-N}\right)$, $\text{N}{\text{O}}_{3}^{-}\text{-N}$ $\left(\text{E-N}{\text{O}}_{3}^{-}\text{-N}\right)$ and soluble organic nitrogen (SON). The contents of exchangeable total nitrogen (ETN), $\text{E-N}{\text{H}}_{4}^{+}\text{-N}$ and $\text{E-N}{\text{O}}_{3}^{-}\text{-N}$ were analysed through the alkaline potassium persulfate digestion–UV spectrophotometric method [23], Nessler's reagent spectrophotometry method [24] and ultraviolet spectrophotometry method [25], respectively. The contents of SON were calculated with the formula $\text{SON}=\text{ETN}-\text{E-N}{\text{O}}_{3}^{-}\text{-N}-\text{E-N}{\text{H}}_{4}^{+}\text{-N}$.

#### 2.3.2. Hydrolysable nitrogen

HN was extracted by adding 40 ml of HCl (6 mol l^−1^) and 2 g of residues of §2.3.1 in a clean centrifuge tube. The tube was sealed and placed in a water bath at 120°C for 24 h. The mixture was centrifuged at 5000 r.p.m. for 15 min, and the supernatant was filtered through a 0.45 µm filter membrane. The filtrate was transferred to a porcelain crucible. The residue was cleaned with ultrapure water several times and the filtrates were transferred into the same porcelain crucible. The residue was freeze dried and kept for RN extraction in the next step. The filtrate was placed in a water bath (120°C) to clean HCl, and the pH was adjusted to 7.5 ± 0.2. HN was composed of ammonium nitrogen (AN), amino acid nitrogen (AAN), amino-sugar nitrogen (ASN) and hydrolysable unidentified nitrogen (HUN). The contents of hydrolysable total nitrogen (HTN), AN, AAN and ASN were analysed through the alkaline potassium persulfate digestion–UV spectrophotometric method [23], Nessler's reagent spectrophotometry method [24], ninhydrin colorimetric method and Elson–Morgen method [22], respectively. The amount of HUN was equal to that of acid HTN minus the sum of AN, ANN and ASN.

#### 2.3.3. Residual nitrogen

RN was extracted by adding H_2_SO_4_ and an accelerator in the residues of §2.3.2, and the concentration was analysed using an automatic Kjeldahl apparatus (Foss 2300, Swiss).

### 2.4. Nitrogen diffusion flux calculation

The diffusion flux of NH_4_^+^–N at the sediment–water interface was calculated using Fick's first law of diffusion [26,27]

2.1 $$F=-\phi D_{S}\frac{\partial c}{\partial x},$$

where *F* stands for the flux of $\text{N}{\text{H}}_{4}^{+}\text{-N}$ (mg m^−1^ d^−1^), and a positive *F* value shows the release of a substance from the sediment to water and vice versa; *φ* stands for the porosity of the surface sediment (0–5 cm); ∂c/∂x is the concentration gradient across the sediment–water interface, which was calculated from the concentration difference of NH_4_^+^-N contents in interstitial and overlying water; and *D*_s_ is the diffusion coefficient for $\text{N}{\text{H}}_{4}^{+}\text{-N}$ in the sediments, which can be calculated from *φ* (*D*_s_ = *φD*_0_, if *φ* < 0.7; *D*_s_ = *φ*^2^*D*_0_, if *φ* > 0.7) as suggested by Ullman and Aller [26]. Here, *D*_0_ is the ideal diffusion coefficient for $\text{N}{\text{H}}_{4}^{+}\text{-N}$ (10^−6^ cm^2^ s^−1^), which is correlated with bottom temperature as follows [28]:

2.2 $$D_{0}\;=\;19.8+0.40\;(T-25).$$

### 2.5. Waterlogged incubation experiment

Waterlogged incubation was applied to investigate the nitrogen mineralization potential in the studied sediments. The waterlogged incubation procedure followed the method of Ouyang *et al.* [29]. The prepared dry sediment samples (2 g) were submerged in 40 ml of distilled water in a series of 100 ml polyethylene centrifuge tubes (acid washed). The tubes were capped and incubated at (40 ± 1)°C in a constant-temperature incubator. The sediment samples were extracted on days 0, 1, 3, 5, 7, 9, 12 and 15. The tubes were oscillated at 25°C in an orbital shaker at 220 r.p.m. for 2 h and centrifuged at 7000 r.p.m. for 15 min. The solutions were placed in 250 ml volumetric flasks. Then, 40 ml of KCl (2 mol l^−1^) was added to the tubes, which were then centrifuged at 7000 r.p.m. for 15 min. The supernatants were placed in the volumetric flasks as well. The latter process was repeated twice with distilled water instead of KCl. The supernatants were also placed in the same volumetric flasks. The solutions in the volumetric flasks were diluted to 250 ml with distilled water and filtered through 0.45 μm GF/C filter membranes. The filtrate was obtained for DTN, $\text{N}{\text{H}}_{4}^{+}\text{-N}$ and $\text{N}{\text{O}}_{3}^{-}\text{-N}$ analyses.

Nitrogen mineralization is mainly controlled by the microorganism and determined by enzyme kinetics. This process fits first-order chemical reaction principles; therefore nitrogen mineralization can be described by the first-order reaction kinetics model. The single first-order exponential model [30] is

2.3 $$N_{t}=N_{0}\;(1-{\text{e}}^{-k_{0}t}),$$

where *N*_t_ is the cumulative mineral N (mg N kg^−1^ of sediment) at time *t* (d), *N*_0_ is defined as PMN (mg N kg^−1^ sediment) and *k*_0_ is the mineralization rate constant (d^−1^).

The net nitrogen mineralization rate was calculated as the content of net cumulative MN divided by the days from the last extraction.

### 2.6. Statistical analysis

For each sediment sample, all determinations of the above experiments were performed in triplicate and expressed as average values with a relative error lower than 5%. Statistical analyses of the mean value and s.d. were performed using SPSS 19.0 software. A spatial distribution map of the sampling sites was drawn with ArcGIS 10.2. Spatial distributions maps of the nitrogen forms and the changes in ON in the studied sediments with time during incubation were created with Origin 9.0. Correlation analyses were conducted between *N*_0_ and different ON fractions and between NH_4_^+^-N release flux and contents of exchangeable inorganic nitrogen (IN) by using Origin 9.0. The single first-order exponential model of nitrogen mineralization dynamics and multiple stepwise regression analysis of different ON forms to *N*_0_ were also fit with Origin 9.0.

## 3. Results and analysis

### 3.1. Spatial distribution of nitrogen fractions in surface sediment

**Figure 2.**
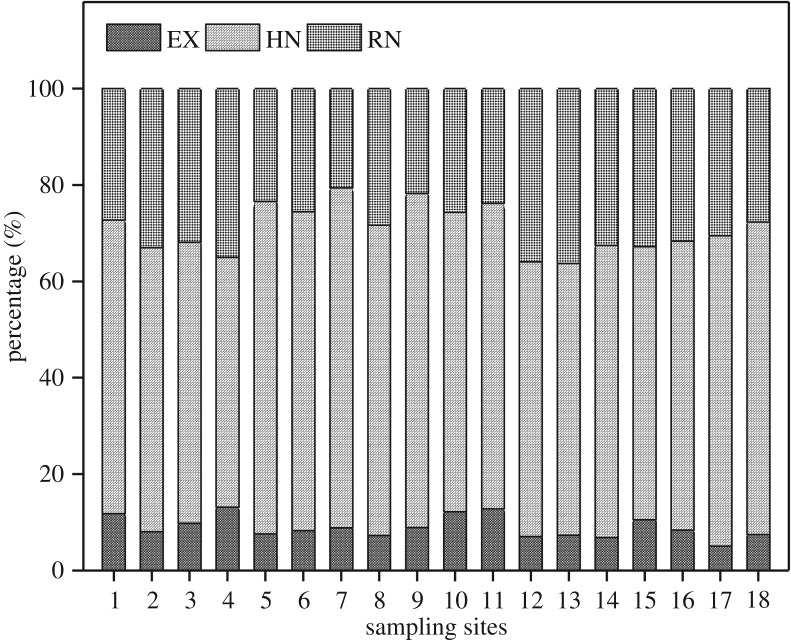
Relative percentages of different nitrogen fractions in the studied sediments.

TN in the studied sediments ranged from 1295.75 to 6151.69 mg kg^−1^. The mean value (3709.32 mg kg^−1^) was between the values of the lowest effect level and the severe effect level regulated in the sediment quality evaluation standard set by Ontario Ministry of Environment and Energy [18]. The contents of TN and different nitrogen fractions were high in the sediments, and the s.d. of EN, HN, RN and TN was 136.22, 631.15, 497.59 and 1197.17, respectively. As shown in figure 2, the contributions of different nitrogen fractions to TN were in the order of HN > RN > EN.

**Figure 3.**
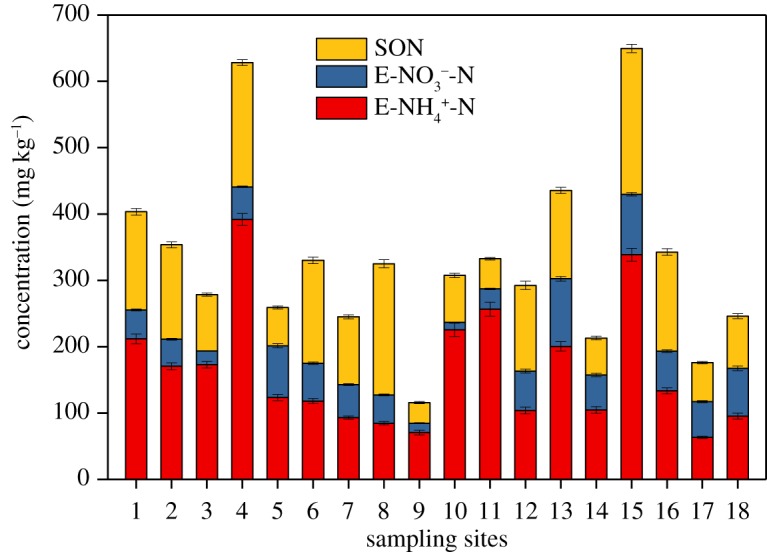
Spatial distribution of EN in the studied sediments.

EN ranged from 115.85 to 649.56 mg kg^−1^ and accounted for 5.05–13.15% of TN. Although EN took up the lowest percentage of TN, it was the predominant form of the bioavailable nitrogen and might contain pore water nitrogen. EN can be used directly by primary productivity in lakes. Hence, it can participate easily in nitrogen cycling and is crucial for nitrogen cycling in sediment. The relative contribution of the three fractions to EN was of the order of $\text{E-N}{\text{H}}_{4}^{+}\text{-N}>\text{SON}>\text{E-N}{\text{O}}_{3}^{-}\text{N}$ (figure 3). $\text{E-N}{\text{H}}_{4}^{+}\text{-N}$ was the main fraction of EN and ranged from 63.47 to 391.78 mg kg^−1^, accounting for an average of 48.92% of EN. $\text{E-N}{\text{O}}_{3}^{-}\text{-N}$ had the lowest concentration and accounted for only an average of 16.85% of EN because the relatively anaerobic environment of sediments weakened the nitrification. SON ranged from 31.08 to 219.69 mg kg^−1^ and accounted for 34.23% of EN.

**Figure 4.**
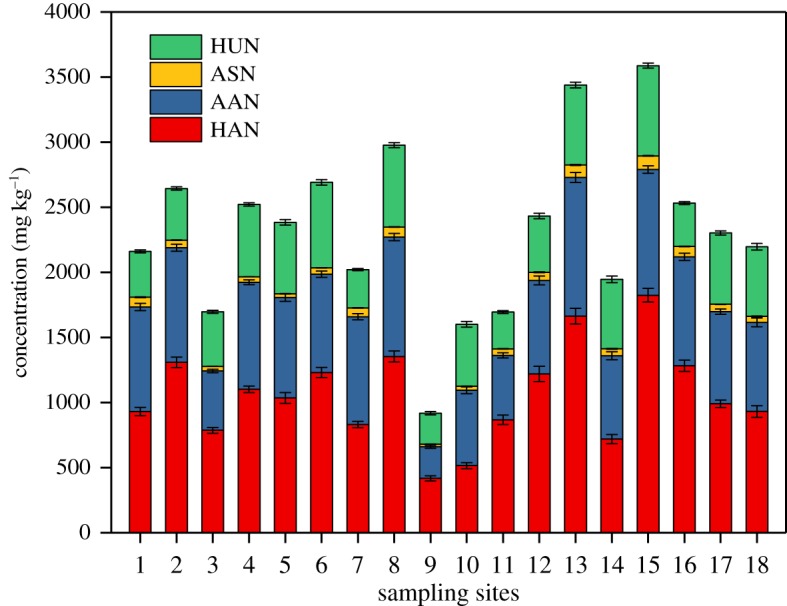
Spatial distribution of HN in the studied sediments.

HN is the main ON fraction in sediment and can be easily mineralized into IN and used by plankton in lakes. HN in the studied sediments ranged from 898.36 to 3482.97 mg kg^−1^ and accounted for 51.87–70.57% of TN. The mean value was 61.97%. The contents of the four fractions of HN was in the order of AN > AAN > HUN > ASN (figure 4). AN was the dominant fraction of HN. It ranged from 243.21 to 1065.11 mg kg^−1^ and accounted for an average of 44.97% of HN. Sediment characteristics (e.g. grain size composition, content of organic matter (OM) and cation exchange capacity) influence the AN content [13]. AN can easily be mineralized into IN. AAN was the second major form of HN; it ranged from 243.21 to 1065.11 mg kg^−1^ and accounted for 31.63% of HN. As a part of AAN, free amino acid can be assimilated directly by microorganisms [31]. ASN is primarily derived from bacterial and fungal cell walls but not from plant cells [32,33]. The content of ASN was the lowest and only accounted for 2.45% of HN. HUN ranged from 237.72 to 2151.45 mg kg^−1^, with an average value of 1118.05 mg kg^−1^. HUN is a hydrolysis-resistant nitrogen form in sediments. Little is known about its origin, and its nature and characterization are unclear [34]. HUN may occur as a structural component of a molecule, such as a bridge constituent linking quinine groups together, or it may be referred to as heterocyclic or aromatic ring-bound nitrogen in humic polycondensates [35].

RN ranged from 281.55 to 2151.45 mg kg^−1^, and accounted for 20.57–36.29% of TN. RN in sediments mainly exists in nitrogen-containing organic heterocyclic molecules or some ON that abound in heterocyclic or aromatic bonds. RN is mainly derived from humic compositions with high condensation degrees. Thus, RN is deemed as a relatively stable fraction with low bioavailability.

### 3.2. Comparison of nitrogen pollution characteristics in sediments of Keluke Lake and other lakes

To further reveal the nitrogen pollution characteristics in the sediments of Keluke Lake, the basic information, TN OM and ON of the lake were compared with those of other typical lakes in China and other countries (table 1).

**Table 1.** Basic information and nitrogen pollution loads of Keluke Lake and other lakes in China and other countries.

region	lakes	surface areas (km^2^)	mean depth (m)	natural trophic status^a^	TN (mg kg**^−^**^1^)	OM (%)	ON (mg kg**^−^**^1^)	references
Switzerland	Lake Zurich	67.3	max. 137	mesotrophic	6000	1.8–3.8	—	[36]
Switzerland	Lake Rotsee	0.46	9	eutrophic	5000–10 000	4.3–6.8	—	[37]
Florida, USA	Lake Apopka	125	1.6	hyper eutrophic	2931.03–31 818.18	34–35	—	[38]
New Hampshire and Maine, USA	Great Bay Estuary	11–23	2	—	2300–2700	2.0–2.6	—	[39]
Canada	Lake Winnipeg	23 750	12	eutrophic	9551–12 322	1.81–2.24	2253–2583	[7]
Japan	Lake Biwa	674	48	oligotrophic–mesotrophic	200–2300	1–2	—	[40]
Japan	Lake Hachiro	48.3	22	eutrophic	160–3010	—	—	[41]
China	Taihu Lake	2338	0.89	light eutrophic	912.03	1.05	743.53	[42,43]
China	Chao Lake	760	3	hyper eutrophic	1424	3.05	1249.16	[44]
China	Dianchi Lake	298	4.4	middle eutrophic	3515.60	4.47	3018.39	[45,46]
China	Poyang Lake	3210	8.4	mesotrophic	1281.56	3.12	1182.35	[47]
China	Dongting Lake	2691	6.7	mesotrophic	1371.85	2.06	1223.66	[22,48]
China	Qinghai Lake	4635	19.15	oligotrophic	2364.78	—	2074.83	[49]
China	Basongcuo Lake	25.9	—	oligotrophic	856.37	—	731.072	[49]
China	Namucuo Lake	1920	—	oligotrophic	3262.22	—	2949.92	[49]
China	Yangzhuoyongcuo Lake	621	30–40	oligotrophic	1890.20	—	1526.09	[49]
China	Keluke Lake	58.6	4∼5	mesotrophic	3709.32	14.29	3493.33	this paper

^a^The trophic statuses of lakes were rated according to the method of Wang *et al.* [50].

The content of TN in the sediments of Keluke Lake was the highest among the compared lakes in China. The difference between the trophic statuses of Keluke Lake and Dianchi Lake is interesting. Dianchi Lake is middle eutrophic, whereas Keluke Lake is mesotrophic, even the contents of TN of the two lakes were close. The TN content in Keluke Lake was also higher than Taihu Lake and Chao Lake, which had a status of light eutrophic. The TN in Keluke Lake was 4.3 times that in Basongcuo Lake, which belongs to the same lake area in China. The TN in Keluke Lake was lower than that of Lake Zurich in Switzerland, Lake Rotsee, Lake Apopka and Lake Winnipeg, and higher than the other compared lakes in other countries.

The content of ON in sediments of Keluke Lake was the highest among the compared lakes. ON was the main fraction of TN in sediments for all of the compared lakes in China, and the ratio of ON to TN ranged from 80.7 to 94.2%. The ratio of ON to TN of Keluke Lake was the highest.

The OM in Keluke Lake was also highest among the other compared lakes except for Lake Apopka.

### 3.3. Changes in inorganic nitrogen in sediments during mineralization

**Figure 5.**
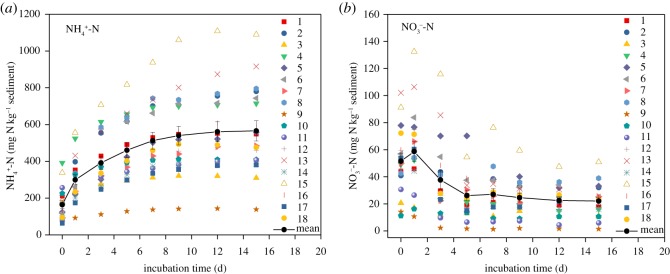
(*a*,*b*) Changes in IN in the studied sediments with time during incubation. The bars indicate the standard error.

In the mineralization process, ON is mineralized into IN by microorganisms. The change in MN in the studied sediments during incubation is shown in figure 5. $\text{N}{\text{H}}_{4}^{+}\text{-N}$ was the main mineralization product in the waterlogged incubation. $\text{N}{\text{H}}_{4}^{+}\text{-N}$ increased from 164.38 to 566.03 mg kg^−1^. The changes in $\text{N}{\text{H}}_{4}^{+}\text{-N}$ in the studied sediments during incubation are similar to the results of Yang *et al*. [51], and the process could be divided into three phases: fast increase phase, transition phase and gentle increase phase [52]. The first day was the fast increase phase, and the net nitrogen mineralization rate was 135.55 mg kg^−1^ sediment d^−1^. The transition phase began on the second day onward, and the mean rate was 30.06 mg kg^−1^ sediment d^−1^, which declined by nearly three times. The nitrogen mineralization rate continually decreased as the incubation proceeded. On the 15th day, which was the gentle increase phase, the rate was only 1.73 mg kg^−1^ sediment d^−1^, which showed a decrease of about 78.5 times compared with the value on the first day.

$\text{N}{\text{O}}_{3}^{-}\text{-N}$ was much lower than $\text{N}{\text{H}}_{4}^{+}\text{-N}$ and displayed an opposite changing trend compared with the latter. $\text{N}{\text{O}}_{3}^{-}\text{-N}$ had a slight increase on the first day, decreased on the third day and stabilized on the seventh day.

### 3.4. Changes in organic nitrogen in sediments during mineralization

**Figure 6.**
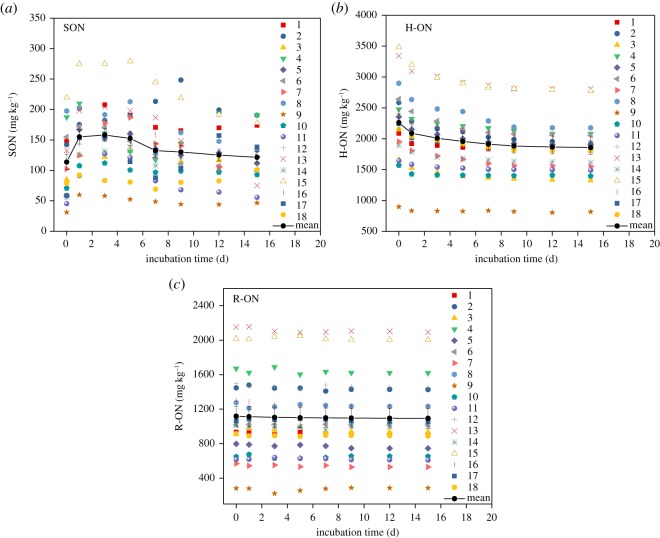
(*a*–*c*) Changes in ON in the studied sediments with time during incubation. The bars indicate the standard error.

During the incubation, SON and hydrolysable organic nitrogen (H-ON) changed significantly over time, whereas residual organic nitrogen (R-ON) did not (figure 6).

The change process of SON can be divided into three phases: increase, decrease and steady phases. In the increase phase, the content of SON increased from 113.71 to 158.09 mg kg^−1^ in the first three days, with a net change rate of 14.79 mg kg^−1^ d^−1^. In the decrease phase, SON decreased from the fifth day, and the net change rate was −5.60 mg kg^−1^ d^−1^. In the steady phase, SON was nearly stable starting from the 12th day and the net change rate was −1.23 mg kg^−1^ d^−1^.

H-ON continually decreased during the incubation, and the change process could be mainly described in two phases: rapid and slow-steady phases. In the rapid phase, the average content of H-ON decreased from 2261.55 to 2008.38 mg kg^−1^ in the first three days, with a net decrease rate of 84.39 mg kg^−1^ d^−1^. The percentage of H-ON in TN decreased from 61.0 to 54.3%. In the slow-steady phase, the average content decreased from 1957.24 to 1856.71 mg kg^−1^ and extended to the end of the incubation with a net rate of 10.05 mg kg^−1^ d^−1^. The percentage of H-ON in TN ranged from 50.7 to 52.9%. Furthermore, a significant positive correlation was observed between SON and H-ON from the third day onward, which meant that the initial SON had been already mineralized on the first or second day and that its ultimate content was mainly controlled by H-ON from the third day on.

The content of R-ON changed slightly and ranged from 1093.30 to 1118.05 mg kg^−1^ during the incubation. The percentage of R-ON in TN was maintained at around 29.8%.

### 3.5. Analysis of nitrogen mineralization potential

The mineralization and immobilization of ON in the sediment layer can provide a source of IN for a water body, which is important to the nitrogen cycling of aquatic ecosystems. The released IN is available for uptake by phytoplankton, which increases the risk of eutrophication [53]. The single first-order exponential model used in this study showed a good fit based on the *R*^2^ values for the equations (table 2). The values of *N*_o_ varied considerably in the tested sediment samples. *N*_o_ ranged from 72.72 to 812.92 mg N kg^−1^ of sediments and the average value was 408.76 mg N kg^−1^ of sediments.

**Table 2.** Estimated parameters of nitrogen mineralization dynamics by the single first-order exponential model based on 15-day incubation.

sampling sites	mineralization formula	*N*_0_	*k*_0_	*R*^2^
1	*y* = 338.56(1−exp(−0.39*x*))	338.56	0.39	0.959**
2	*y* = 582.43(1−exp(−0.36*x*))	582.43	0.36	0.947**
3	*y* = 142.87(1−exp(−0.49*x*))	142.87	0.49	0.945**
4	*y* = 314.94(1−exp(−0.43*x*))	314.94	0.43	0.971**
5	*y* = 453.76(1−exp(−0.21*x*))	453.76	0.21	0.973**
6	*y* = 655.08(1−exp(−0.24*x*))	655.08	0.24	0.967**
7	*y* = 384.48(1−exp(−0.30*x*))	384.48	0.3	0.940**
8	*y* = 686.40(1−exp(−0.41*x*))	686.4	0.41	0.976**
9	*y* = 72.72(1−exp(−0.32*x*))	72.72	0.32	0.977**
10	*y* = 181.31(1−exp(−0.68*x*))	181.31	0.68	0.921**
11	*y* = 164.33(1−exp(−0.16*x*))	164.33	0.16	0.961**
12	*y* = 372.22(1−exp(−0.28*x*))	372.22	0.28	0.969**
13	*y* = 711.65(1−exp(−0.23*x*))	711.65	0.23	0.943**
14	*y* = 290.49(1−exp(−0.31*x*))	290.49	0.31	0.974**
15	*y* = 812.92(1−exp(−0.21*x*))	812.92	0.21	0.965**
16	*y* = 481.38(1−exp(−0.23*x*))	481.38	0.23	0.939**
17	*y* = 315.19(1−exp(−0.30*x*))	315.19	0.3	0.970**
18	*y* = 396.92(1−exp(−0.34*x*))	396.92	0.34	0.971**

*y*: cumulative mineral N; *x*: incubation time; **significant at *p* < 0.01.

To identify the main sources of mineralizable ON in sediment, a correlation analysis was conducted between PMN and different ON fractions, including F-ON, E-ON, AN, AAN, ASN, HUN and RN, in the studied sediments (table 3). The contents of SON, AN, AAN, ASN, HUN and RN were all significantly and positively correlated with PMN, which meant that all of these ON fractions might contribute to the nitrogen mineralization process. Meanwhile, the contribution proportions might differ because the correlation coefficients between *N*_0_ and different ON fractions varied. Furthermore, multi-collinearity always exists between different independent variables in multiple regression analysis, which results in an unreasonable explanation of the partial regression coefficient to directly establish linear regression equations between the dependent and independent variables. To further recognize the relative contribution of different ON forms to *N*_0_, multiple stepwise regression analysis was used to establish the optimal regression equation. The result was PMN = 0.53AN−152.83 (*n* = 18, *R*^2^ = 0.826, *p* < 0.01). Thus, AN was the most effective contributor to mineralizable ON, and the potential mineralization ability of sediment nitrogen was mainly influenced by AN.

**Table 3.** Coefficients of correlation between PMN and SON, AN, AAN, ASN, HUN and RN in sediments of Keluke Lake.

ON fractions	correlation equation (*n* = 18)	*R*^2^
SON	*y* = 0.20*x* + 32.44	0.557**
AN	*y* = 1.55*x* + 420.97	0.826**
AAN	*y* = 0.81*x* + 402.06	0.750**
ASN	*y* = 0.08*x* + 24.59	0.542**
HUN	*y* = 0.43*x* + 296.79	0.450**
RN	*y* = 1.70*x* + 423.10	0.524**

*y*: *N*_0_; *x*: content of relevant ON fraction; **significant at *p* < 0.01.

### 3.6. Analysis of nitrogen release potential

The sediment–water interface is an important boundary for substance exchange between sediments and the overlying water in shallow lakes. Nutrient flux across the interface is crucial for nutrient cycling and primary productivity maintenance in water ecosystems. For most contaminated sediments, the nutrient concentrations in interstitial water are usually higher than those in overlying water, and the release of nutrients from sediments to overlying water is mainly caused by the concentration gradient [54,55]. The release process can be accelerated by sediment re-suspension, which is likely to occur in shallow lakes. In this study, the $\text{N}{\text{H}}_{4}^{+}\text{-N}$ diffusion flux ranged from 24.14 to 148.75 mg m^−2^ d^−1^, with a mean value of 70.85 ± 34.17 mg m^−2^ d^−1^. The positive values of the flux indicated that $\text{N}{\text{H}}_{4}^{+}\text{-N}$ was released from the sediments to the overlying water. Hence, the sediments in Keluke Lake served as an internal source of nitrogen.

**Figure 7.**
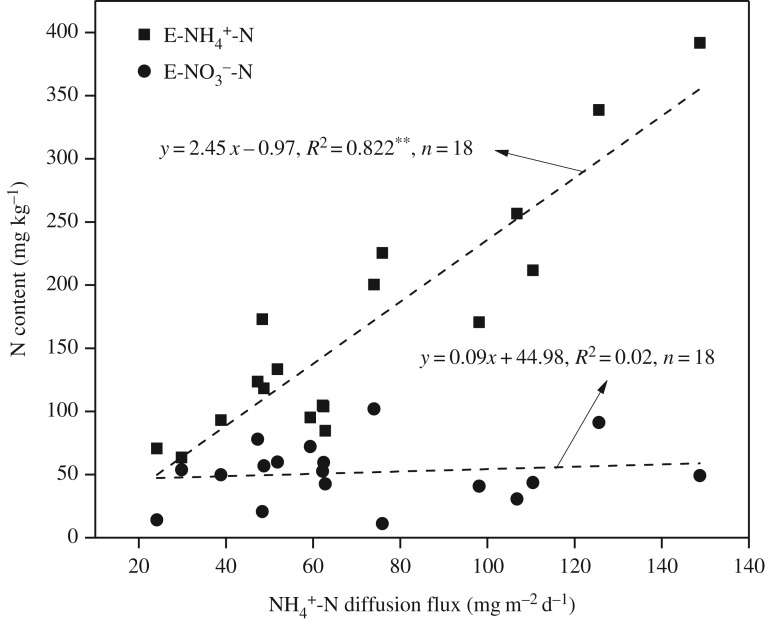
Relationship between $\text{N}{\text{H}}_{4}^{+}\text{-N}$ diffusion flux and exchangeable IN in sediments of Keluke Lake. **significant at *p* < 0.01.

To further examine the influence of bioavailable nitrogen fractions on nitrogen release from the sediments, the correlation between the $\text{N}{\text{H}}_{4}^{+}\text{-N}$ release flux and the contents of exchangeable IN was analysed. The $\text{N}{\text{H}}_{4}^{+}\text{-N}$ diffusion flux was significantly correlated with $\text{E-N}{\text{H}}_{4}^{+}\text{-N}$, but not with $\text{E-N}{\text{O}}_{3}^{-}\text{-N}$ (figure 7). Thus, the nitrogen release potential from sediments to overlying water was mainly influenced by $\text{E-N}{\text{H}}_{4}^{+}\text{-N}$.

## 4. Discussion

TN in sediments of Keluke Lake reached the polluted level, and the content was highest among the compared lakes in China including the most famous three eutrophic lakes, namely Taihu Lake, Chao Lake and Dianchi Lake. The contents of Keluke Lake and Dianchi Lake were close, but their eutrophic states were different. Although the two lakes are positioned on plateaus, their climate, socio-economic and sediment conditions differ. Keluke Lake is located in a cold temperate zone, with the average annual temperature of 3.9°C [56]. Dianchi Lake is located in a subtropical zone, and the mean annual temperature is 14.5 to 17.8°C. A high water temperature is beneficial to nutrient release from sediments to overlying water. The population in Dianchi Lake Basin in 2015 was 3.632 million people [57], which is much higher than the 0.0733 million of Keluke Lake Basin. Hence, the pollution load from human activities is higher in Dianchi Lake than in Keluke Lake. Furthermore, the content of OM in the sediments of Keluke Lake was 14.29%, which is 3.2 times of that in Dianchi Lake. A high OM can absorb large amounts of nitrogen and causes nitrogen deposit in sediments.

The TN content in Keluke Lake was also higher than Taihu Lake and Chao Lake, which had a status of light eutrophic. This can also be attributed to the differences in socio-economic, climate and sediment conditions. The differences of TN and OM between Keluke Lake and Lake Zurich in Switzerland can be attributed to the larger water depth of Lake Zurich compared with Keluke Lake.

One important reason for the high TN in sediments of Keluke Lake can be attributed to high OM, for sediments with high OM can easily absorb nitrogen. The OM of Keluke Lake mainly comes from the sheep and cow dung carried by Bayin River. Many aquatic plants exist in the lake, and they are deposited in the sediments after their death, which can also contribute OM. In addition, the lake only has inlet water and nearly no outlet water. Hence, most of the nitrogen in inflows is accumulated in the sediments. The sediments in Keluke Lake act as a nitrogen pool, especially for ON, and can affect the water quality. The nitrogen release potential must be evaluated to thoroughly understand the impacts of sediments on water quality.

ON becomes available to aquatic organisms only after it has been mineralized [14,45,58]. The transformation of nitrogen via the immobilization–mineralization process is essential for cycling and bioavailability as well as for understanding the biogeochemical cycle of nitrogen in lake ecosystems. PMN is the most active part of the ON pool in sediments and is an important nitrogen source to aquatic ecosystems [14]. PMN can be released into the overlying water via the sediment–water interface and can thus affect the trophic status of lakes. A high PMN content in sediments indicates great potential N release capability due to the high amount of N mineralized [14,45]. The PMN in the sediments of Keluke Lake ranged from 72.72 to 812.92 mg N kg^−1^ of sediment, with a mean value of 408.76 mg N kg^−1^ of sediment. The PMN in Keluke Lake was higher than that in Taihu Lake (176.208–329.748 mg N kg^−1^) [58], Poyang Lake and Hongze Lake but lower than that in Xuanwu Lake, Yue Lake [14] and Dianchi Lake (1154.76) [45]. The PMN of sediments in Keluke Lake was higher than that in most of the typical shallow lakes in the middle and lower reaches of the Yangtze River area in China and showed a relative higher mineralization potential. Furthermore, the mean $\text{N}{\text{H}}_{4}^{+}\text{-N}$ diffusion flux across the sediment–water interface of Keluke Lake was 70.85 mg m^−2^ d^−1^, which was higher than that of Chaohu Lake (39.60 mg m^−2^ d^−1^) [59], Taihu Lake (34.1 mg m^−2^ d^−1^) [60], Dianchi Lake (30.18 mg m^−2^ d^−1^) [58], Dongting Lake (16.23 mg m^−2^ d^−1^) [22] and Baiyangdian Lake (12.3 mg m^−2^ d^−1^) [61], thereby showing a relatively higher release capability.

During the waterlogged incubation experiment, $\text{N}{\text{H}}_{4}^{+}\text{-N}$ and SON were the main mineralized nitrogen form, and their contents increased. The changes of $\text{N}{\text{H}}_{4}^{+}\text{-N}$ are similar to those of Dou *et al*. [62] and can be explained by substrate availability and microbial characteristics. In the fast increase phase, the readily available organic material was degraded by ‘zymogeneous’ microbes accompanied with the consumption of available organic material and accumulation of $\text{N}{\text{H}}_{4}^{+}\text{-N}$, resulting in the restriction of ammonifiers' activity. Then, during the transition and gentle increase phases, many resistant organic materials were degraded by the ‘autochthonous’ group of microbes. SON was easier to mineralize compared with the other ON fractions. The initial SON in the sediments was quickly mineralized at the beginning of incubation, and its content should be reduced theoretically. However, SON only accounted for an average of 3% of TN in the studied sediments. HN accounted for an average of 61.0% of TN and was the main contributor to nitrogen mineralization. During the mineralization process, HN and other ON were initially transformed into SON and then into IN. Thus, the content of SON increased. The mineralized $\text{N}{\text{H}}_{4}^{+}\text{-N}$ and SON can be released into the overlying water and used by aquatic organisms. $\text{N}{\text{H}}_{4}^{+}\text{-N}$ can be directly absorbed and used by planktons. Studies have shown that 10–80% of SON can be used by aquatic organisms. The release of $\text{N}{\text{H}}_{4}^{+}\text{-N}$ and SON from sediments to overlying water can promote the growth of algae and increase the risk of lake eutrophication [63].

The concentration of $\text{N}{\text{O}}_{3}^{-}\text{-N}$ showed an opposite changing trend to $\text{N}{\text{H}}_{4}^{+}\text{-N}$ during the waterlogged incubation experiment. It is because, at the beginning of the waterlogged incubation, the activity of nitrobacteria was high and part of the ON in the sediments was disintegrated by nitrobacteria into $\text{N}{\text{O}}_{3}^{-}\text{-N}$. Hence, $\text{N}{\text{O}}_{3}^{-}\text{-N}$ increased on the first day. As the cultivation time went by, the oxygen in the tube was consumed, and the activity of nitrobacteria was restrained. Other microorganisms also expended $\text{N}{\text{O}}_{3}^{-}\text{-N}$, so cumulative $\text{N}{\text{O}}_{3}^{-}\text{-N}$ decreased.

Keluke Lake is the largest freshwater lake in Qaidam Basin and plays an important role in the local ecological environment, social economy and culture. However, the lake has demonstrated declining water quality and ecological degradation in recent years. The sediments in Keluke Lake pose a potential threat to lake water quality. The sediment release risk will further increase if global warming and human disturbance intensify, and the threat posed by sediments to water quality will be aggravated. To effectively protect Keluke Lake, polluted sediments should be comprehensively considered and treated by appropriate measures, such as environmental dredging, phytoremediation and adding passivators. The total content, speciation, mineralization of ON and the release flux at sediment–water interface should be considered comprehensively to evaluate the effects of nitrogen in sediments to water quality. The occurrence, migration and transformation characteristics of phosphorus in sediments should be also studied to further reveal the role of sediments in a freshwater lake.

## 5. Conclusion

The forms, mineralization and release potential nitrogen were studied to reveal the occurrence characteristics and potential impacts of nitrogen in sediments on the water quality of Tibetan Plateau freshwater lakes. The nitrogen load in Keluke Lake sediments was larger than that in most of the compared lakes in China and abroad and was presented as a nitrogen sink. ON was the dominant fraction, accounting for 94.2% of TN. The mean values of PMN and $\text{N}{\text{H}}_{4}^{+}-\text{N}$ diffusion flux were higher than those of most typical shallow lakes in the middle and lower reaches of the Yangtze River in China, and they showed a relatively higher mineralization and release potential. The sediments pose a potential risk for water quality and should be governed by applying appropriate measures, such as environmental dredging, phytoremediation and adding passivators.

## Supplementary Material

Supporting Data 1

## Supplementary Material

Supporting Data 2

## Data accessibility

The datasets supporting this article have been provided as the electronic supplementary material. All data for our work are presented in tables 1–3.
